# Tetanus development during hospitalization in an older adult

**DOI:** 10.1016/j.idcr.2024.e02114

**Published:** 2024-11-09

**Authors:** Naoki Okawa, Naoto Hosokawa

**Affiliations:** Department of Infectious Diseases, Kameda Medical Center, 929 Higashi-cho, Kamogawa, Chiba 296-8602, Japan

**Keywords:** Diabetic foot lesions, Dysphagia, Necrotizing soft tissue infection, Tetanus, Tetanus toxoid, Trismus

## Abstract

Tetanus is a fatal infectious disease caused by neurotoxins produced by *Clostridium tetani*. Currently, tetanus has no confirmatory tests; it is often diagnosed based on clinical signs and symptoms. Due to vaccination, tetanus is uncommon in developed countries; however, it remains an important differential diagnosis, especially in older adults. Herein, we report the case of an older adult who developed tetanus during the course of his disease although no findings indicated tetanus on admission. Thus, tetanus can develop during hospitalization, and if symptoms such as dysphagia or difficulty opening the mouth occur, tetanus should be considered as a clinical diagnosis.

## Highlights

● Tetanus can occur during hospitalization.
● Diabetic foot lesions are high-risk wounds for tetanus.
● Tetanus vaccination should be considered in these patients with diabetic foot lesions.

## Introduction

Tetanus is an acute illness characterized by muscle spasms and autonomic nervous system dysfunction caused by exotoxins produced by the spore-forming bacterium *Clostridium tetani* [1], [2]. Unvaccinated people are at the highest risk for tetanus. In developed countries, its inclusion in routine childhood vaccination programs has reduced tetanus incidence [3]. Occasional cases of tetanus are reported, mostly in older adults. The reason is that they may have been born before vaccination programs were introduced or that vaccine-induced antibody titers decline over time [4]. Other risk factors include diabetes, immunosuppression, and intravenous drug use. According to the Centers for Disease Control and Prevention, persons with diabetes accounted for 14 % of all reported tetanus cases from 2000 to 2019 [2].

Usually, tetanus diagnosis is made according to typical clinical findings. Tetanus often starts with risus sardonicus and masseter rigidity[5]. Thus, patients experience symptoms such as an inability to open the mouth and difficulty swallowing and breathing. The incubation period ranges from 3 to 21 days, averaging 8 days. However, a longer incubation period exceeding 1 month has been reported [6]. Generally, duration varies by injury site; injuries away from the central nervous system have a longer incubation period. In this report, we present the case of an older patient who developed tetanus during his disease course, with no findings suggestive of tetanus on admission.

## Case presentation

A 63-year-old man with fever and right leg pain for 1 week was brought to our hospital’s emergency department. He had a history of type 2 diabetes mellitus and had voluntarily ceased insulin therapy for half a year. He also had undergone amputation of his right second toe. He lived barefoot and wore sandals, with his feet contaminated with soil. He had no history of contact with animals. On his right plantar area, a month-old ulcer that started as an abrasion was noted. He had no history of intravenous drug use and a history of incomplete tetanus immunization.

**Fig. 1:**
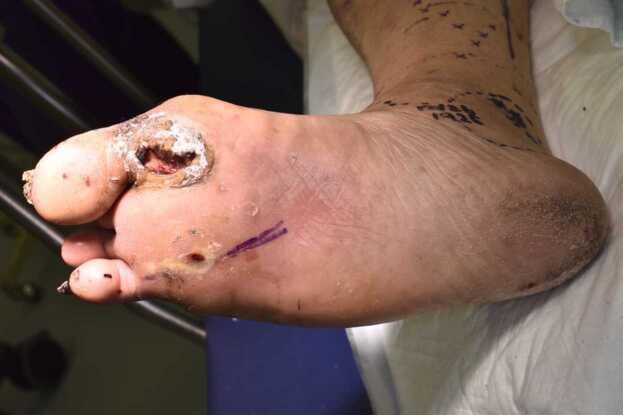
Ulcers on the right plantar surface measured 2 × 3 cm wide and 1 cm deep, exhibiting erythema, warmth, tenderness, and swelling.

**Fig. 2:**
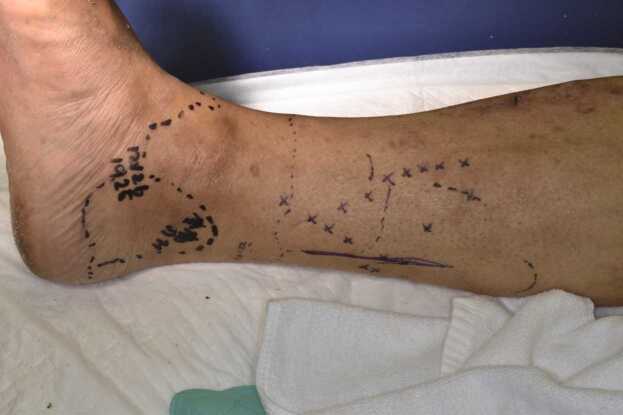
Erythema, warmth, tenderness, and swelling were observed distal to the black dotted line on the ankle. The same observation was noted in the medial condyle surrounded by the black dotted line, except for tenderness. The lower leg was mildly warm with no tenderness or erythema. The blue marks and lines are the markers made by the surgeon for the operation.

**Fig. 3:**
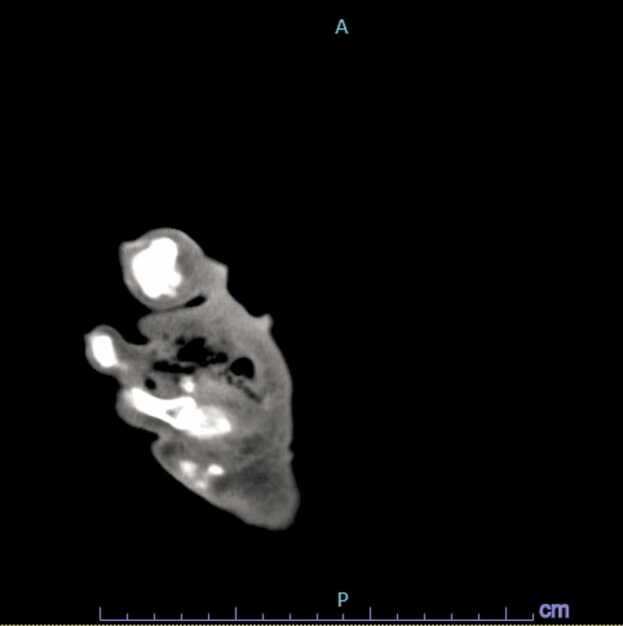
Computed tomography scan of the lower extremity showing subcutaneous gas on the right plantar surface.

Physical examination revealed that the patient was stable, with clear consciousness. The baseline vital signs were as follows: temperature, 38.5 °C; blood pressure, 178/121 mmHg; pulse rate, 114 beats/min; respiratory rate, 21 breaths/min; and oxygen saturation, 97 % on room air. His cardiopulmonary and abdominal examination results were normal. The ulcers on the sole of his right foot measured 2 × 3 cm wide and 1 cm deep (Fig. 1). His lower extremities and plantar surfaces exhibited erythema, warmth, tenderness, and swelling (Fig. 2). Blood tests showed normal platelet count and hemoglobin levels but revealed an elevated white blood cell count (12,700/μL). The results of liver and kidney function tests, blood electrolyte levels, and chest radiography were normal, but his glycosylated hemoglobin was high (10.5 %). Furthermore, computed tomography of the lower extremities revealed subcutaneous gas on the right plantar surface (Fig. 3).

**Fig. 4:**
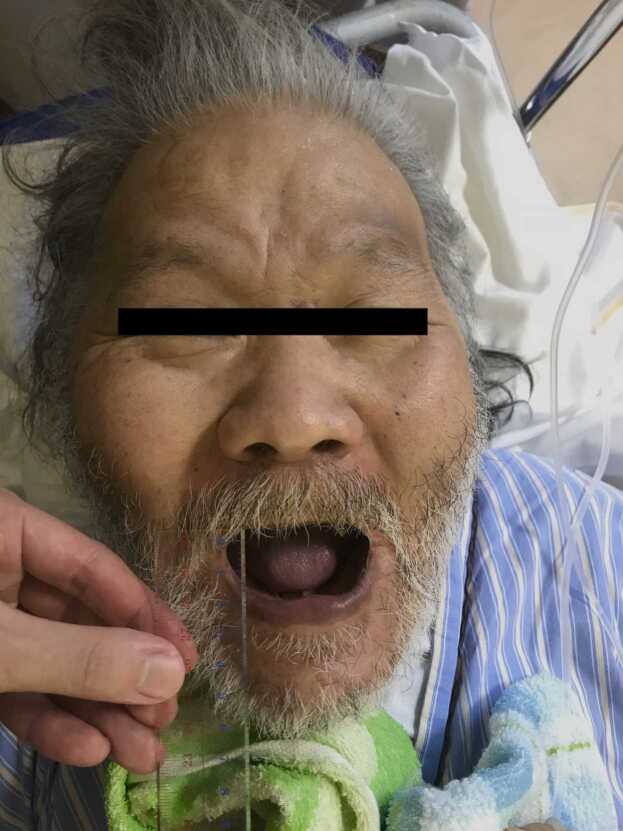
A picture of lockjaw.

On admission, we initially suspected a necrotizing soft-tissue infection caused by diabetic foot lesions. The surgeon made exploratory incisions on the right plantar surface and lower extremities and considered debridement or amputation to be not needed. Samples from the wound were then sent for aerobic and anaerobic bacterial culture. He was treated with vancomycin, piperacillin/tazobactam, and clindamycin. After treatment was initiated, symptoms in the right lower extremity improved. Wound cleansing and debridement were continued. The wound culture showed the growth of *Proteus vulgaris*, *Streptococcus anginosus*, *Enterococcus faecalis*, and *Staphylococcus lugdunensis*, but no *C. tetani* growth was observed. Subsequently, vancomycin and clindamycin were discontinued according to the susceptibility results and clinical course. On the same day, he found swallowing difficult. On day 9, dysphagia symptoms worsened. Choking episodes were experienced while eating, but were relieved by back blows. On day 10, he developed difficulty opening his mouth, indicating trismus (lockjaw) (Fig. 3), and neck stiffness. Hence, he was clinically diagnosed with tetanus. Human tetanus immunoglobulin 4500 IU and tetanus toxoid were then administered. He was admitted to the intensive care unit, where endotracheal intubation was required. Midazolam, diazepam, and propofol were administered for sedation to manage the muscle spasms, and continuous magnesium sulfate to control autonomic dysfunction. In addition, blood pressure was controlled using continuous labetalol. Then, sedation was gradually withdrawn, and no seizures occurred. The intubation lasted for 13 days. Fig. 4.

After receiving piperacillin/tazobactam for a total of 54 days for osteomyelitis in the second, third, and fourth metatarsal heads identified on magnetic resonance imaging, he was discharged home.

## Discussion

An older patient with diabetic foot gangrene was hospitalized for a necrotizing soft-tissue infection but developed tetanus during his hospitalization despite having no symptoms suggestive of tetanus on admission. The clinical course of the patient highlights two important points: (1) the onset of tetanus symptoms after admission and (2) diabetic foot lesions as high-risk tetanus wounds.

The most interesting aspect of this patient’s clinical course is the appearance of tetanus symptoms on the fifth day after admission despite the absence of findings suggesting tetanus on admission. Therefore, *C. tetani* could be infecting the patient simultaneously with the bacteria causing the necrotizing soft-tissue infection. Although *C. tetani* was not detected in anaerobic cultures of the wound, anaerobic cultures are known to be frequently negative. In addition, a positive culture of *C. tetani* from wounds is not useful for diagnosis because it does not indicate whether the organism contains the toxin-producing plasmid [5]. Few cases of tetanus after admission have been reported, but given the incubation period of tetanus, the disease may develop during hospitalization. In one case, a man in his 70 s developed tetanus on the seventh day of hospitalization for pressure ulcers and pneumonia [7]. Postoperative tetanus has also been reported, although rarely [8]. Epidemiologic data indicate that 0–3.5 % of reported cases of tetanus occur postoperatively, most commonly after intraperitoneal surgery [9]. Both exogenous and endogenous sources are responsible for postoperative tetanus. Owing to the decline in tetanus incidence, clinicians in developed countries may be less familiar with its clinical presentation; especially if it occurs during hospitalization, diagnosis may be even more difficult. Tetanus often starts with risus sardonicus and masseter rigidity, in this case, dysphagia was the first manifestation of tetanus. There are some reports that dysphagia is the first symptom of tetanus [10]. Thus, clinicians should consider tetanus in inpatients with a consistent clinical presentation of dysphagia and poor vaccination history.

Tetanus is a vaccine-preventable disease. However, in Japan, where the diphtheria-tetanus- pertussis vaccine was introduced as a routine vaccine in 1968, many older people have not been vaccinated. Tetanus is a reportable disease in Japan, and most of the affected patients are older adults who have not received routine immunization [11]. In other developed countries, most cases of tetanus also occur in older adults [12]. Increasing the vaccination rates, especially among older adults, is critical to reduce tetanus incidence. Therefore, aggressive tetanus toxoid vaccination of old-aged patients is recommended as part of routine practice. Furthermore, chronic diabetic foot ulcers are one of the most common complications of diabetes and are susceptible for *C. tetani* invasion, causing tetanus infection [13]. However, the foot care section of the American Diabetes Association’s 2024 Standards of Care in Diabetes and the International Working Group on the Diabetic Foot/Infectious Diseases Society of America Guidelines on the Diagnosis and Treatment of Diabetes-related Foot Infections (IWGDF/IDSA 2023) have no mention of tetanus vaccination [14], [15]. Because diabetic foot lesions are high-risk wounds for tetanus, tetanus vaccination should be more strongly considered in these patients, and in some cases, both tetanus toxoid and immunoglobulin may be required. Tetanus prophylaxis is not only an important consideration in acute traumatic wound evaluation but should also be considered as a routine practice in the management of chronic wounds including diabetic foot ulcers [16].

## Conclusion

Tetanus can develop during hospitalization, even without tetanus symptoms at initial presentation. Therefore, initiating appropriate treatment as early as possible is crucial when symptoms of suspected tetanus develop. Furthermore, diabetic foot lesions are at a high risk for tetanus development; thus, tetanus prophylaxis should be given greater consideration in these patients.

## CRediT authorship contribution statement

**Naoto Hosokawa:** Supervision. **Naoki Okawa:** Writing – review & editing, Writing – original draft, Methodology, Investigation, Formal analysis, Data curation, Conceptualization.

## Ethical approval

Ethics approval was not required.

## Consent

Written informed consent was obtained from the patient for the publication of this case report.

## Funding

No funding was obtained for this study.

## Conflict of Interest

The authors declare no conflict of interest.

## References

[1] Yen L.M., Thwaites C.L. (2019). Tetanus. Lancet 2019.

[bib2] 2Centers for Disease Control and Prevention. Tetanus.[https://www.cdc.gov/tetanus/ 2024/06/06 Accessed]

[3] Our World in Data. Tetanus [https://ourworldindata.org/tetanus 2024/06/06 Accessed].

[4] National Institute of Infectious Diseases. National Epidemiological Surveillance of Vaccine- Preventable Disease. Age distribution of tetanus antibody positives in Japan, 2018. [https://www.niid.go.jp/niid/en/y-graphs.html 2024/06/06 Accessed].

[5] Birch T.B., Bleck T.P. Tetanus (Clostridium tetani). Mandell, Douglas, and Bennett’s Principles and Practice of Infectious Diseases 244:2948–2953.e2.

[6] Saltoglu N., Tasova Y., Midikli D., Burgut R., Dündar I.H. (2004). Prognostic factors affecting deaths from adult tetanus. Clin Microbiol Infect.

[7] Yuya O., Hiroki T. (2022). Is it necessary the prevention tetanus for the case of pressure ulcer? A case of tetanus during hospitalization due to pressure ulcer and pneumonia. KANTO J Jpn Assoc Acute Med.

[8] Katz K.C., Walmsley S.L. (2000). Postoperative tetanus: a case report. CMAJ.

[9] Dhalla S. (2004). Postsurgical tetanus. Can J Surg.

[10] Okada T., Yabe H., Ando R., Iwaki H., Nishikawa N., Nagai M., Sei H., Nishida N., Nomoto M. (2015). Case Report; A case of tetanus with dysphagia as an initial symptom. Nihon Naika Gakkai Zasshi.

[11] Nakajima M., Aso S., Matsui H., Fushimi K., Yasunaga H. (2018). Clinical features and outcomes of tetanus: analysis using a National Inpatient Database in Japan. J Crit Care.

[12] Filia A., Bella A., von Hunolstein C., Pinto A., Alfarone G., Declich S., Rota M.C. (2014). Tetanus in Italy 2001–2010: a continuing threat in older adults. Vaccine.

[13] Hoseini Tavassol Z., Sajjadpour Z., hasani-Ranjbar S., Pejman Sani M., Aghaei Meyboodi H., Larijani B. (2022). Do patients with diabetic foot ulcer need booster dose of tetanus vaccine?. J Diabetes Metab Disord.

[14] American Diabetes Association Professional Practice Committee (2024). 12. Retinopathy, Neuropathy, and Foot Care: Standards of Care in Diabetes-2024. Diabetes Care.

[15] Senneville E., Albalawi Z., van Asten S.A., Abbas Z.G., Allison G., Aragon-Sanchez J., Embil J.M., Lavery L.A., Alhasan M., Oz O., et al. IWGDF/IDSA guidelines on the diagnosis and treatment of diabetes-related foot infections (IWGDF/IDSA 2023) Diabetes Metab Res Rev. 2023;40:e3687. 10.1002/dmrr.3687 37779323

[16] Farnworth E., Roberts A., Rangaraj A., Minhas U., Holloway S., Harding K. (2012). Tetanus in patients with chronic wounds - are we aware?. Int Wound J.

